# Controlling A_*x*_Mn[Fe(CN)_6_] charge transfer pathways through tilt-engineering for enhanced metal-to-metal interactions†

**DOI:** 10.1039/d4ma00262h

**Published:** 2024-08-31

**Authors:** A. Regueiro, J. Castells-Gil, C. Shen, I. Mikulska, C. Allen, L. Bogani, R. Torres-Cavanillas

**Affiliations:** a Instituto de Ciencia Molecular, Universitat de València Catedrático José Beltrán 2 46980 Paterna Spain Ramon.torres@uv.es; b School of Chemistry, University of Birmingham Birmingham B15 2TT UK; c Department of Materials, Oxford University 21 Banbury Rd Oxford OX2 6NN UK lapo.bogani@materials.ox.ac.uk; d Diamond Light Source, OX11 0DE, Didcot Didcot Oxfordshire OX11 0DE UK; e Diamond Light Source, electron Physical Science Imaging Centre OX11 0DE Didcot UK

## Abstract

The induction of structural distortion in a controlled manner through tilt engineering has emerged as a potent method to finely tune the physical characteristics of Prussian blue analogues. Notably, this distortion can be chemically induced by filling their pores with cations that can interact with the cyanide ligands. With this objective in mind, we optimized the synthetic protocol to produce the stimuli-responsive Prussian blue analogue A_*x*_Mn[Fe(CN)_6_] with A = K^+^, Rb^+^, and Cs^+^, to tune its stimuli-responsive behavior by exchanging the cation inside pores. Our crystallographic analyses reveal that the smaller the cation, the more pronounced the structural distortion, with a notable 20-degree Fe–CN tilting when filling the cavities with K^+^, 10 degrees with Rb^+^, and 2 degrees with Cs^+^. Moreover, this controlled distortion offers a means to switch on/off its stimuli-responsive behavior, while modifying its magnetic response. Thereby empowering the manipulation of the PBA's physical properties through cationic exchange

## Abstract 2

We explored the interplay between structure and metal-to-metal charge transfer in A_*x*_Mn[Fe(CN)_6_], incorporating K, Rb, and Cs. Activation/deactivation of the intramolecular charge transfer is influenced by cation-induced structural tilting.

## Graphical abstract

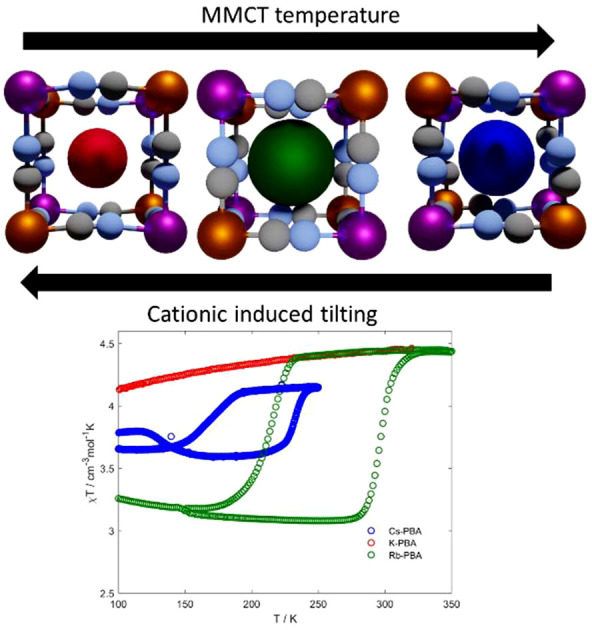

## 1. Introduction

Stimuli-responsive materials, working in environmental conditions stand at the forefront of contemporary technological needs. These materials hold immense promise across various applications, including memory storage, mechanical actuation, sensing, and biomedicine, among others.^1,2^ Their versatility lies in their ability to undergo reversible changes in physical properties triggered by external stimuli like mechanical stress, temperature variations, light exposure, or electric fields.^3,4^ Among these compounds, the Prussian blue analogue (PBA) with formula Rb_*x*_Mn^II^[Fe^III^(CN)_6_]_(2+*x*)/3□ (1−*x*)/3_ (where □ denotes a vacancy), 0 ≤ *x* ≤ 1, has emerged as an exceptionally promising stimuli-responsive material. The PBA may exhibit a fascinating phenomenon known as metal-to-metal charge transfer (MMCT) between Fe and Mn, triggered by temperature or light.^5–7^ This transfer produces a reversible switching between a high-temperature phase (HT), characterized by Mn^II^ (*S* = 5/2)–Fe^III^ (*S* = 1/2), and a low-temperature phase (LT), with Mn^III^ (*S* = 2)–Fe^II^ (*S* = 0). Remarkably, this electron transfer can occur with a memory effect around room temperature. Previous studies have demonstrated that the amount of Rb included in the structure has a strong influence on the transition temperatures and the width of the hysteresis loop.^8^ As a guiding principle, higher Rb composition correlates with higher transition temperatures and narrower hysteresis. Classically, this has been attributed to the fact that, in the absence of any counterion, the structural defects are required to balance the different charges of Mn^2+^ and [Fe(CN)_6_]^3−^, leading to a ratio Fe/Mn of 0.67. Thus, the counterion is expected to play an innocent role only acting as a medium to obtain a perfect structure, balancing the charges, and enabling the MMTC to occur for a ratio of Fe/Mn ≥ 0.86.^8,9^

However, it is important to remark that the cation within the PBA's cavity is not a passive component, as it can induce structural distortions in the cubic lattice, altering their physical and chemical properties.^10,11^ This has been demonstrated in A⊂{[Fe(Tp)(CN)_3_)]_4_[Co(pzTp)(CN)_3_)]_4_} with A being K, Rb, or Cs; or in the extended A_2_Co_4_[Fe(CN)_6_]_3.3_, among others.^12^

This distortion is triggered by the tilting of the M-cyanides coordination polyhedral due to electrostatic interaction with the A counterion.^13^ This action reduces the unit cell volume and brings the transition metal closer. Consequently, the smaller the A cation, the greater its charge density and thus, the electrostatic distortion. This phenomenon, termed tilt-engineering, has gathered significant attention in battery research, as these structural changes upon cation lattice incorporation can impact the PBA's mechanical stability, seriously affecting their capacity retention.^14,15^ Moreover, this counteranion-mediated chemical tilting offers a direct avenue to manipulate the magnetic or electrical properties of PBAs. For instance, consider the A_2_Mn^II^[Mn^II^(CN)_6_] PBA, where the critical temperature (*T*_c_) decreases as the size of the A counterion increases from Na to Cs, with *T*_c_ tripling for the smaller A cation.^16–18^ This seemingly counterintuitive trend arises from an increase in the exchange coupling between Mn atoms as the Mn–N–C bond deviates from linearity. Thus, the bent cyanide bridges play a crucial role in the superexchange mechanism by intensifying the coupling through the shortening of Mn^II^⋯Mn^II^ atomic distances. Unfortunately, despite the high potential of PBA's octahedral tilting as a tool to modulate magnetic properties, and in particular, as a tool to trigger spin phenomena, it has remained underexplored. Herein, we aim to face this issue, by synthesizing the PBA A_*x*_Mn[Fe(CN)_6_]_(2+*x*)/3(1−*x*)/3_ using different cations (A = K, Rb, or Cs), to explore the structural distortion produced by the A cation and its correlation with their magnetic behaviour, using this structural tilting to activate/deactivate the MMCT in this particular PBA.

## 2. Experimental section

### Synthetic protocols

All chemical reagents were purchased and used without further purification. Potassium chloride, rubidium chloride, cesium chloride, manganese(ii) chloride, and potassium hexacyanoferrate were purchased from Sigma-Aldrich. Ultrapure water (18.2 MΩ) was used in the following syntheses.

#### Synthesis K_*x*_-PBA

2.5 mL of an MnCl_2_ aqueous solution 0.1 M was added drop by drop to 2.5 mL of an aqueous solution of K_3_[Fe(CN)_6_] 0.1 M and a variable amount of KCl, ranging from 0 M (K_0.06_-PBA), 0.2 M (K_0.2_-PBA), 0.5 M (K_0.4_-PBA), 1 M (K_0.5_-PBA), 2 M (K_0.7_-PBA). After addition, the solution is stirred for 1 h. The precipitate is filtered and washed with MilliQ H_2_O three times to remove the excess of precursors. After drying in the air, a brown precipitate is obtained.

#### Synthesis Rb_*x*_-PBA

2.5 mL of an MnCl_2_ aqueous solution 0.1 M was added drop by drop to 2.5 mL of an aqueous solution of K_3_[Fe(CN)_6_] 0.1 M and a variable amount of RbCl, ranging from 0.1 M (Rb_0.4_-PBA), 0.2 M (Rb_0.5_-PBA), 0.5 M (Rb_0.6_-PBA), 1 M (Rb_0.7_-PBA). After addition, the solution is stirred for 1 h. The precipitate is filtered and washed with MilliQ H_2_O three times to remove the excess of precursors. After drying in the air, a brown precipitate is obtained.

#### Synthesis Cs_*x*_-PBA

2.5 mL of an MnCl_2_ aqueous solution 0.1 M was added drop by drop to 2.5 mL of an aqueous solution of K_3_[Fe(CN)_6_] 0.1 M and a variable amount of CsCl, ranging from 0.1 M (Cs_0.8_-PBA), 0.2 M (Cs_0.9_-PBA), 0.5 M (Cs-PBA), 1 M (Cs_1.2_-PBA). After addition, the solution is stirred for 1 h. The precipitate is filtered and washed with MilliQ H_2_O three times to remove the excess of precursors. After drying in the air, a brown precipitate is obtained.

### Physical characterization

Scanning electron microscopy and energy dispersive spectroscopy studies were carried out on a Hitachi S4800 microscope. Transmission electron microscopy studies were carried out on a Jeol 300 arm microscope operating at 80 kV. Samples were prepared by dropping suspensions on lacey formvar/carbon copper grids (300 mesh). Attenuated total reflectance Fourier-transform infrared spectra were collected in an Agilent Cary 630 FTIR spectrometer in the 4000–500 cm^−1^ range in the absence of KBr pellets. Magnetic data were collected with a Quantum Design MPMS XL-5 susceptometer equipped with a SQUID sensor. DC FC magnetization measurements were performed under an applied magnetic field of 100 Oe. Powder-X-ray diffraction measurements were carried out in 0.5 mm glass capillaries mounted and aligned in a PANanlytical Empyrean diffractometer using Cu Kα radiation (Cu Kα = 1.5418 Å) with a PIXcel detector, operating at 40 mA and 45 kV. Profiles were collected in the 8° < 2*θ* < 70° range with a step size of 0.013°. In the case of the Cs_*x*_-PBA samples for Rietveld refinements, PXRD patterns were collected on a STOE STADI P diffractometer using Mo Kα1 radiation (0.70926 Å). Analysis of the PXRD patterns *via* Rietveld refinement was carried out using the software TOPAS Academic v6. ^19^Mn and Fe K-edge X-ray absorption spectroscopy (XAS) data were collected at B18 Core-XAS beamline^19,20^ at Diamond Light Source (experiment number sp38973-1). XAS spectra were recorded in transmission mode using double-crystal monochromator equipped with Si(111) crystals and using Pt coated collimating and focusing mirrors. Elimination of higher energy harmonics has been achieved by using double-mirror harmonic rejection system. Measurements have been done at room temperature. Thermogravimetric analysis were carried out by a Mettler Toledo TGA/SDTA 851*e* model operating in the 25–400 °C range. K, Rb, Cs, Mn, and Fe contents were determined using an inductively coupled plasma mass spectrometry (ICP-MS) Agilent 7900 after the digestion of the powder in an acidic medium at 180 °C.

## 3. Results and discussion

**Fig. 1:**
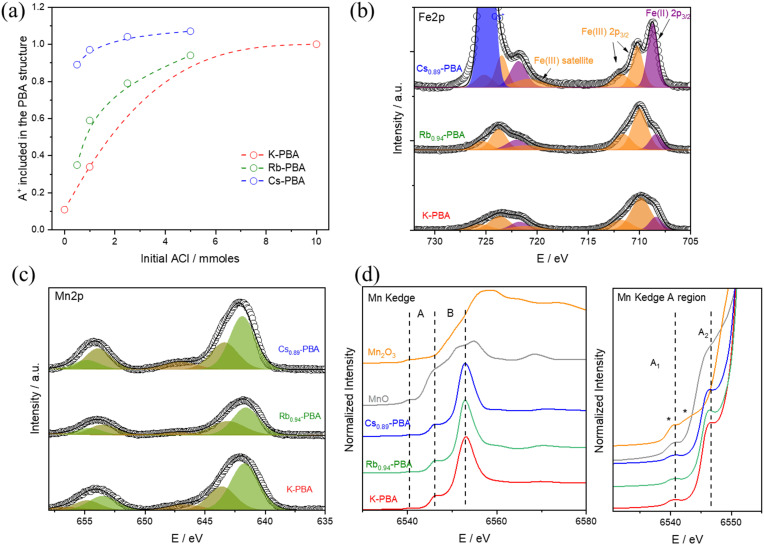
(a) ICP-MS quantification of A+ included in the A_*x*_-PBAs depending on the initial ACl concentration. (b) and (c) Fe and Mn 2p XPS region of K-PBA, Rb_0.94_-PBA, and Cs_0.89_-PBA. (d) Mn K-edge XANES spectra of the same samples and MnO and Mn_2_O_3_ as Mn(ii) and (iii) references. Complete XANES region at the left, and zoom of the pre-edge region at the right.

To synthesise the A_*x*_Mn[Fe(CN)_6_]_(2+*x*)/3□(1−*x*)/3_ complexes (hereinafter referred to as A_*x*_-PBA, with A denoting K, Rb, or Cs) with the A cation appropriately included in the structure, a MnCl_2_ solution is added at 0.3 mL h^−1^ over a K_3_[Fe(CN)]_6_ and ACl solution. It should be noted that the delayed addition is essential to slow down the synthetic kinetics, allowing the A counterion to properly fill the PBA cavity and produce large microcrystals, Fig. S1–S3 (ESI†). For the Rb_*x*_-PBA it is well known that *x* increases with the starting RbCl concentration.^9^ However, this has not been studied for the K_*x*_-PBA and Cs_*x*_-PBA. As a result, firstly, we synthesized the three compounds by adding various amounts of KCl, RbCl, and CsCl, aiming to find the optimal synthetic conditions for preparing all of the A-PBAs with a comparable structure (for more information see experimental section). All of the samples yield a brown powder, whose morphology was characterized by scanning electron microscopy (SEM), composition by energy dispersive spectroscopy (EDS), induced coupled plasma mass spectrometry (ICP-MS) and thermogravimetric analysis (TGA), Tables S1, S2 and Fig. S4 (ESI†). The oxidation state of the metals was roughly estimated by Fourier transform infrared spectroscopy (FT-IR), and later for the samples K-PBA, Rb_0.94_-PBA, and Cs_0.89_-PBA deeply determined by X-ray photoemission spectroscopy (XPS) and X-ray absorption near edge structure (XANES). Our results show that spherical-like microparticles (Fig. S1, ESI†) with the chemical formula K_0.11_Mn[Fe(CN)_6_]_0.71_ were obtained in the absence of any additional ACl (according to ICP-MS, Table S1 and S2, ESI†). However, as we increased the initial amount of ACl, the concentration of the A cation in the PBA structure also increased (Fig. 1a and Tables S1, S2, ESI†).

Interestingly, this change led to the formation of cubic particles instead of the spherical ones (Fig. S1–S3, ESI†). This suggests that having more A cations enhances the crystallinity, possibly by enabling the obtention of Fe/Mn ratios closer to 1 (refer to Tables S1 and S2, ESI†). Despite this, it's important to note that the average particle size remained largely unchanged (Fig. S5a, ESI†). Remarkably, for Cs_*x*_-PBA, mixing an equimolar amount of precursors yields a PBA with *x* = 0.89. In contrast, to achieve Rb_*x*_-PBA with *x* = 0.94 and K_*x*_-PBA with *x* = 1, a tenfold and twentyfold excess of ACl need to be added, respectively. Based on this, we can infer that as the size of the A cation increases, its incorporation into the PBA structure becomes easier. As shown in Fig. 1b–d, all K_*x*_-PBA, Rb_*x*_-PBA, and Cs_*x*_-PBA compositions primarily contain Mn^II^ and Fe^III^ metals. It's important to note that the stretching vibrations of cyanide are highly sensitive to the oxidation states of Mn and Fe, making FT-IR an ideal technique for their identification. In all cases, a prominent peak at 2169 cm^−1^, corresponding to Mn^II^–NC–Fe^III^, and a smaller band at 2080 cm^−1^, associated with Mn^II^–NC–Fe^II^ impurities, are observed (Fig. S6, ESI†).^21^ While these impurities are more common in Rb_*x*_-PBA and Cs_*x*_-PBA, Mn^II^–NC–Fe^III^ remains predominant in all three A_*x*_-PBAs except in Cs_1.07_-PBA.^22^

Finally, we verified that the A-site cations are integrated into the structure rather than simply being adsorbed onto the particle surface. This was accomplished by comparing the experimental powder X-ray diffraction (PXRD) patterns with simulated spectra and by Fe K-edge EXAFS analysis (Fig. S13 and Table S9, ESI†). By examining the changes in peak intensities of PXRD patterns, we confirmed that the cavities are indeed filled with the different A counterions (Fig. S7–S9, ESI†). However, we noted significant discrepancies in relative intensities between the experimental and simulated PXRD in the Cs samples. This is due to the different arrangement of Cs ions inside the cavities, which significantly impacts the relative intensities (Fig. S9, ESI†)

Once we determined the optimal conditions to fabricate the different A_*x*_-PBAs, we focused our attention on the compounds K-PBA, Rb_0.94_-PBA, and Cs_0.89_-PBA. We decided to study in detail these three because (1) in all cases the ratio A:Mn is over the *x* ≥ 0.6 reported that is required for the obtention of the MMCT;^8^ (2) they exhibit a comparable amount of A^+^, enabling us to properly compare the effect of the A cation in the PBA structure.

**Fig. 2:**
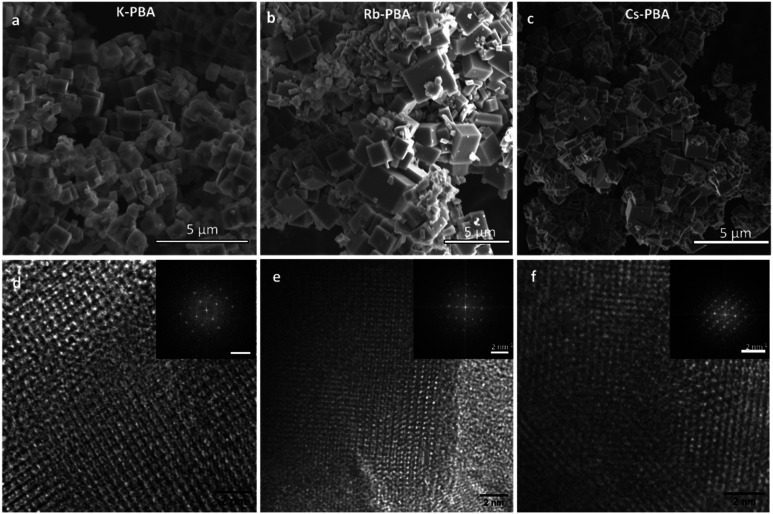
SEM image of the K-PBA (a), Rb_0.94_-PBA (b), and Cs_0.89_-PBA (c) microcrystals. HR-TEM images of the same compounds, K-PBA (d), Rb_0.94_-PBA (e), and Cs_0.89_-PBA (f). In the inset, it is shown the FFT of the HR-TEM images.

Fig. 2a–c depicts cubic microcrystals with lateral diameters of 0.9 ± 0.3 μm, 1.2 ± 0.7 μm, and 0.7 ± 0.4 μm for K-PBA, Rb_0.94_-PBA, and Cs_0.89_-PBA, respectively. As expected, the size of the crystals did not change much depending on the alkaline cation (Fig. S5b, ESI†). On the other hand, due to the exceptional crystallinity of the microcubes we could observe by high-resolution transmission electron microscopy (HR-TEM) the coherent shepherd-check pattern associated with the alternating Mn and Fe atomic array, as well as a cubic structure (Fig. 2d–f). From where the Mn–Fe distances within the cubic lattice could be estimated at roughly 5.1 Å in all cases, agreeing with the predicted Mn^II^–NC–Fe^III^ distance.^23,24^

The oxidation state of the different metals was deeply explored using XPS and XANES. Starting with the XPS results, the three samples exhibit similar Fe 2p and Mn 2p bands, as shown in Fig. 1b–c. In the Fe 2p region, a predominance of Fe(iii) (orange bands) is observed, with some impurities of Fe(ii) (purple bands), which aligns well with the FTIR results. Notably, the Fe(ii) impurities are larger in the Cs_0.89_-PBA sample than in the other samples. Moving to the Mn 2p region, as shown in Fig. 1c, the three compounds exhibit identical spectra. Regarding the Mn oxidation state, due to the presence of multiple splitting and satellite features for Mn(ii) and Mn(iii) atoms, its determination by XPS is not fully reliable. Therefore, we used Mn K-edge XANES, which is a more reliable technique for determining the Mn oxidation state. As depicted in Fig. 1d, the Mn K-edge XANES profile and energy position confirm the absence of Mn(iii) impurities, with only Mn(ii) detected. This is evident in the pre-edge (A) and edge (B) regions. In the lower energy A region, MnO shows a doublet at the same position called A1 and A2, characteristic of Mn(ii). In contrast, Mn_2_O_3_ displays two features marked with asterisks in Fig. 1d at energies lower and higher than A1. Additionally, the main edge position (B) at 6545 eV for the PBA samples appears at the same position as MnO, while it is significantly shifted in Mn_2_O_3_. Therefore, we can conclude: (1) there is no Mn(iii), or if there is any, it is in such a low percentage that it will not have a significant impact on the magnetism of the compound. (2) There is a more significant Mn(ii)–Fe(ii) impurities in the Cs_0.89_-PBA than in the other samples.

**Fig. 3:**
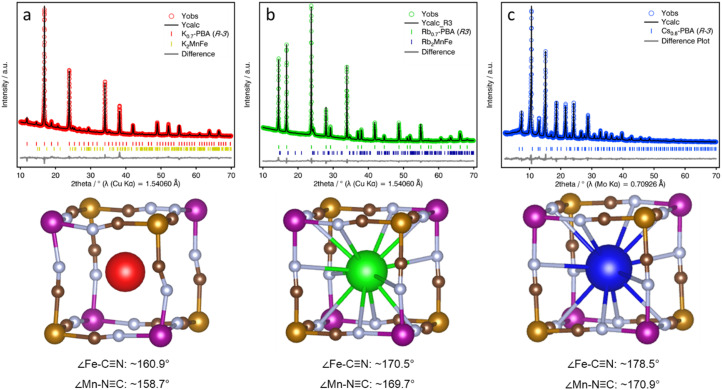
Symmetry-mode Rietveld refinement fits (top) of the PXRD patterns and snapshot of the structural models (bottom) for K-PBA (a), Rb_0.94_-PBA (b), and Cs_0.89_-PBA (c). Colour code: Mn (purple), Fe (ochre), C (brown), N (light blue), K (red), Rb (green), Cs (blue). Structure visualisation was carried out with VESTA.

To explore the structural changes induced by the A-CN electrostatic interaction, we studied the structure of the three A-PBAs by PXRD Rietveld analysis. The Rietveld refinement of the Rb_0.94_-PBA, and Cs_0.89_-PBA agreed well with a cubic face-centered structure, with a formula of Rb_0.95_Mn[Fe(CN)_6_] (*a* = 10.5639(1), *R*_wp_ = 1.58%, *F*4̄3*m*) and Cs_0.93_Mn[Fe(CN)_6_] (*a* = 10.5768(3), *R*_wp_ = 5.97%, *Fm*3̄*m*). In contrast, K-PBA crystallizes in a primitive cubic unit cell with a formula K_0.91_Mn[Fe(CN)_6_]_0.80_ (*a* = 10.5197(2), *R*_wp_ = 2.53%, *Pm*3̄*m*), likely due to the ordering of the Fe vacancies (Fig. S10, and Table S3, ESI†). Remarkably, it was observed that in the case of Rb_0.94_-PBA there is a preference for occupying alternating cavities. However, no occupational A-cation order was found in Cs_0.89_-PBA, and thus it was better modelled in the F*m*3̄*m* space group. Additionally, minor impurities were also observed due to the formation of A_2_Mn^II^[Fe^II^(CN)_6_]. On the other hand, the unit cell parameter increases with the size of the A-site cation which, assuming that the C–N bond length remains invariant, suggests different conformation of the CN groups. This can also be seen in atomic displacement parameters obtained for the CN ligand which vary following the sequence K-PBA > Rb_0.94_-PBA > Cs_0.89_-PBA, further suggesting a higher degree of structural distortion with decreasing the size of the A-site cation. However, although the cubic structural models provide a good description of the experimental XRD patterns (Fig. S10–S12, and Tables S3, S5 and S7, ESI†), confirming they are essentially cubic, the symmetry restrictions imposed by the cubic symmetry do not allow for the proper characterization of the CN ligands' deviation from their ideal positions. Therefore, in order to study the distortion of the CN ligands as a function of the A-site cation, we carried out a symmetry-mode Rietveld refinement using ISODISTORT21 assuming a trigonal distortion (Fig. 3, see Experimental Section for details).^25^ This allowed us to estimate the deviation of the CN ligands from their ideal positions, yielding an average deviation from the ideal 180° ∠Fe–C–N and ∠Mn–N–C bond angle to ***ca.*** 160.9° and 158.7° for the K-PBA, respectively. This deviation decreases in the case of Rb_0.94_-PBA with bond angles of 170.5° (∠Fe–C–N) and 169.7° (∠Mn–C–N), and it is further reduced to 178.5° (∠Fe–C–N) and 170.9° (∠Mn–C–N), for Cs_0.89_-PBA. In all cases, the larger deviation is observed for the ∠Mn–C–N angle, as it has been observed previously for A_2_Mn[Mn(CN)_6_] (A = Na, K, Rb) PBAs.^16–18^ This trend is likely due to the charge density of the A-site cation, as smaller cations, like K^+^, may provide greater electrostatic interaction with the negatively charged CN ligands, leading to larger distortions. A similar effect has been observed in the high-pressure behaviour between AMn[Co(CN)_6_] (A = Rb, Cs) PBAs, where smaller Rb^+^ cations lower the critical pressure for its first structural transition from cubic (*F*4̄3*m*) to a distorted tetragonal (*P*4̄*n*2) phase by an order of magnitude.^26^

This structural distortion was also explored by more localized crystallographic techniques. In particular, extended X-ray absorption fine structure (EXAFS) spectroscopy and pair distribution function (PDF) analysis. EXAFS experiments at the Fe K edges were conducted to measure the changes in Mn–Fe distances influenced by local structural distortions induced by different cations, Fig. S13 (ESI†). We focused on the Fe K-edge because the Mn K and Fe K edges rise at 6539 eV and 7112 eV, respectively. Being separated only by ∼570 eV, the Mn K-edge EXAFS spectra are limited in the *k*-range to 11 Å^−1^, which is insufficient to obtain precise information about the local structure around Mn atoms. Structural parameters of the nearest neighbours of Fe atoms are listed in Table S9 (ESI†). Unfortunately, due to the large errors obtained during the fitting, all the Fe–Mn distances overlap. This is a consequence of the intrinsic EXAFS limitations for determining longer distances of neighbouring atoms, preventing the proper determination of the Fe–Mn distance (∼5.3 Å) in the different samples with the required precision.^27^ Nevertheless, thanks to the EXAFS treatment, we could also confirm the local inclusion of the A cations inside the cavities (see Table S9, ESI†).

On the other hand, PDF analysis of total scattering is a much more powerful tool in this case, allowing us to explore the M-CN tilting present in the K-PBA and Cs_0.89_-PBA samples locally, given its larger accessible *r* range. Unfortunately, PDF data for the Rb_0.94_-MnFe sample could not be obtained due to the persistent photoluminescence of the Rb atoms caused by the Mo X-ray source used for the PDF data acquisition. Fig. S14 (ESI†) displays the comparison between the PDFs of K-PBA and Cs_0.89_-PBA samples, showing differences in the Fe–Mn distance at approximately 5.3–5.4 Å (see inset), which is larger for the Cs sample. To estimate the extent of the distortion due to the size reduction of the A-site cation, we calculated the average M–C–N angle by considering the average M–C/N and Mn–Fe distances obtained from the PDF. This was done assuming a transverse distortion of the C–N ligands, as previously observed in the Fe[Fe(CN)_6_],^28^ and a C–N bond distance of 1.15 Å. This yielded M–CN angles of 157–161° and 170–180° for K-PBA and Cs_0.89_-PBA, respectively, in good agreement with our XRD analysis.

After the crystallographic characterization, we proceed to take a step further and study this cation induce chemical pressure as an atomic knob to modulate the MMCT and magnetic properties of Mn[Fe(CN)_6_]. Therefore, we proceed to investigate the magnetic response of the three A-PBAs from 2 K to 300 K. All the magnetic information is summarized in Table 1.

**Table 1:** Summary of the magnetic parameters of the K_0.7_-PBA, Rb_0.7_-PBA, and Cs_0.8_-PBA, and their Fe–CN angle

	*T*_LT→HT_ (K)	*T*_HT→LT_ (K)	Δ*T* (K)	*Θ* (K)	*C*_exp_ (cm^−3^ mol^−1^ K)	Fe–CN angle
K-PBA	—	—	—	−4	4.6	160.9°
Rb_0.94_-PBA	296.9	216.5	80.4	6.9	2.9	170.5°
Cs_0.89_-PBA	232.8	169.4	63.4	1.5	3.4	178.5°

**Fig. 4:**
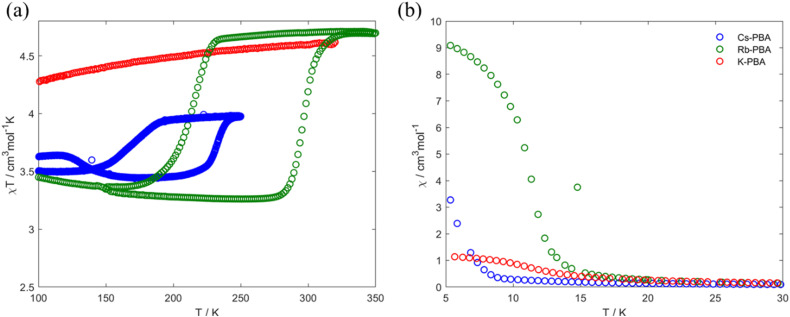
Thermal variation of the *χT* product (a) and *χ* (b) for the K-PBA (red), Rb_0.94_-PBA (blue), and Cs_0.89_-PBA (green).

In the high-temperature region between 100–300 K, Fig. 4a, the Rb_0.94_-PBA and Cs_0.89_-PBA samples exhibit abrupt increases/decreases in *χT* with temperature, whereas the K-PBA does not. For Rb_0.94_-PBA, at 300 K, it displays an experimental *χT* of 4.6 cm^3^ mol^−1^ K, which decreases to 3.2 cm^3^ mol^−1^ K below 216 K before returning to its initial value above 300 K. This is in reasonably good agreement with the switching between the LT (Mn(iii) *S* = 2 and Fe(ii) *S* = 0, theoretical *χT* of 3 cm^3^ mol^−1^ K) and the HT phases (Mn(ii) *S* = 5/2 and Fe(iii) *S* = 1/2, theoretical *χT* of 4.7 cm^3^ mol^−1^ K). The small discrepancy observed in both phases is due to the Mn(ii)–Fe(ii) small impurities observed in XPS and XAS. Similarly, Cs_0.89_-PBA demonstrates analogous behavior, transitioning from 4.0 cm^3^ mol^−1^ K to 3.5 cm^3^ mol^−1^ K below 169 K, then reverting to its initial value at 232 K. The more significant discrepancy between the theoretical and experimental values is also due to the more prominent fraction of Mn^II^–NC–Fe^II^ also observed by XAS (Fig. 1), which is not susceptible to switching its spin state. It is worth mentioning that the MMCT of both compounds exhibits a significant hysteretic behaviour, above the typical thermal hysteresis or other related phase transition molecular materials,^3,4,29^ and in good agreement with previous reports of this type of PBAs.^5–8^

Moving to the low-temperature range (below 100 K), the three compounds exhibit distinct magnetic behaviours, as shown in Fig. 4b. K-PBA remains in the HT phase due to the absence of MMCT, while its Rb and Cs counterparts adopt the LT state. This difference in the metal's oxidation state plays a crucial role in the magnetic interaction between Mn and Fe as shown in Fig. 4b. In the case of K-PBA, it behaves as a ferrimagnet, evidenced by the *χT* decrease with decreasing temperature until reaching a minimum, followed by an abrupt increase (Fig. S16a, ESI†). This aspect is further investigated by analyzing its magnetic response above 10 K according to the Curie–Weiss law. Through this analysis, we found a Curie constant (*C*) of 4.6 cm^3^ mol^−1^ K, closely matching the expected spin-only value for Mn^II^–Fe^III^ (4.7 cm^3^ mol^−1^ K), along with a negative Weiss constant (*Θ*) of −4 K (Fig. S17a, ESI†). The negative *Θ* indicates a short-range antiferromagnetic coupling, in which uncompensated spins lead to the observed ferrimagnetism. On the other hand, Rb_0.94_-PBA (below 220 K) and Cs_0.89_-PBA (below 140 K) exhibit increasing *χT* values with decreasing temperature, which is indicative of a ferromagnetic interaction (Fig. S16b and c, ESI†). This observation is confirmed by fitting the magnetic properties of both materials to the Curie–Weiss law in the LT phase. For Rb_0.94_-PBA, we calculated *C* = 2.9 cm^3^ mol^−1^ K and *Θ* = 6.9 K, while for Cs_0.89_-PBA, *C* = 3.4 cm^3^ mol^−1^ K and *Θ* = 1.5 K, Fig. S17b and c (ESI†). Once again, the deviation between C and the ideal spin-only value of Mn^III^ and Fe^II^ (3.0 cm^3^ mol^−1^ K) is attributed to the presence of residual A_2_Mn^II^[Fe^II^(CN)_6_], while the positive *Θ* confirms a ferromagnetic exchange. The ferromagnetism observed in the LT phase arises from the interaction between Mn–Mn mediated by the diamagnetic Fe^II^. Additionally, the trend of *Θ*(Rb) > *Θ*(Cs) is a consequence of the unit cell expansion upon Cs insertion, separating further the Mn–Mn than in the case of Rb intercalation, thereby reducing the magnetic exchange and shifting the Tc to lower values.

Our research suggests that these different magnetic behaviours and MMCT can be understood through a structural lens, linked to the tilting of Mn and Fe octahedra induced by the A cation. Suggesting that in K-PBA, the significant tilting of the Fe–CN polyhedra (160.9°) deactivates the MMCT, maintaining the HT Mn^II^–Fe^III^ state regardless of temperature. Conversely, for angles above 170°, the charge transfer is reactivated, allowing Rb_0.94_-PBA to switch its spin state at 300 K (from LT to HT phase) and 240 K (from HT to LT phase). Furthermore, when introducing Cs the Mn to Fe angle approaches linearity, shifting the phase transition to lower temperatures, between 220 K (from LT to HT phase) and 140 K (from HT to LT phase). This is because MMCT is a thermally activated phenomenon; hence, linear Mn–CN–Fe angles facilitate charge transfer, requiring less thermal energy to occur. It must also be noted that slight differences in stoichiometry may play a role in the thermal shift. Additionally, the appearance of MMCT allows the ferromagnetic Mn^III^–Fe^II^ LT phase to be reached below 100 K, with the exchange coupling decreasing as the Fe–CN approaches linearity. Furthermore, and adding an extra degree of freedom, we observed that a cation exchange can be carried out post-synthetically. In this context, we manage to exchange the K from the K-PBA for Rb, activating the MMCT post-synthetically (Fig. S18, ESI†). However, it must be noted that the reactivated PBA exhibits a high fraction of compounds that do not present MMCT.

## Conclusions

In this paper, we delve into the study of the chemical pressure induced by the A cation (A = K, Rb, or Cs) in the A_*x*_Mn[Fe(CN)_6_] PBA, which leads to a structural distortion that can be used to modulate its physical properties. We successfully incorporated all three cations into the PBAs with comparable filling. From this study, we concluded that: (1) small cations like K cause significant structural distortion, whereas Rb and Cs induce less distortion. (2) The structural distortion induced by K inhibits the MMCT in this PBA, while Rb and Cs enable it. Furthermore, the cation inside the pore can be exchanged post-synthetically to activate or deactivate MMCT. (3) The chemical pressure induced by the cation also affects the magnetic ordering and critical temperatures in PBAs. This discovery lays the groundwork for designing bistable compounds with switchable capabilities, poised to meet diverse needs ranging from memory storage to biomedicine, batteries, and electrocatalysis.

## Author contributions

A. R. and R. T.-C. were responsible for the design, synthesis and characterization of the different materials presented in this work, and were involved in all the experimental measurements. J. C.-G was in charge of the structural characterization and data interpretation. C. A. was in charge of the Transmission electron microscopy characterization. C. S. contributed to the data interpretation. I. M. performed XAS measurements and EXAFS analysis. R. T.-C. designed the work and were involved in the development and coordination of all the experimental parts, discussion of the results and preparation of the manuscript. L. B. supervised all the work and the preparation of the manuscript. All the authors revised and contributed to the presented manuscript.

## Data availability

All data are available within the article (and its ESI†) and from the corresponding authors upon reasonable request.

## Conflicts of interest

There are no conflicts to declare.

## Supplementary Material

MA-005-D4MA00262H-s001
