# Supplementary material for: Controlling AxMn[Fe(CN)6] charge transfer pathways through tilt-engineering for enhanced metal-to-metal interactions
Source: Mater Adv. 2024 Aug 31;5(18):7473–80. doi: 10.1039/d4ma00262h (PMC11373532; doi:10.1039/d4ma00262h)
Supplement: MA-005-D4MA00262H-s001 [file MA-005-D4MA00262H-s001.pdf]

Supplementary Information (SI) for Materials Advances.
This journal is © The Royal Society of Chemistry 2024

Controlling $A_xMn[Fe(CN)_6]$ Charge Transfer Pathways through Tilt-Engineering for Enhanced Metal-to-Metal Interactions

A. Regueiro,[1] J. Castells-Gil,[2] C. Shen,[3] C. Allen, [3,4] L. Bogani,[3]* R. Torres-Cavanillas[1,3]*

1. Instituto de Ciencia Molecular, Universitat de València, Catedrático José Beltrán 2, 46980, Paterna, Spain
2. School of Chemistry, University of Birmingham, Birmingham B15 2TT
3. Department of Materials, Oxford University, 21 Banbury Rd, Oxford OX2 6NN, United Kingdom
4. Diamond Light Source, electron Physical Science Imaging Centre, OX11 0DE, Didcot, United Kingdom

*Corresponding authors: ramon.torrescavanillas@materials.ox.ac.uk,

lapo.bogani@materials.ox.ac.uk

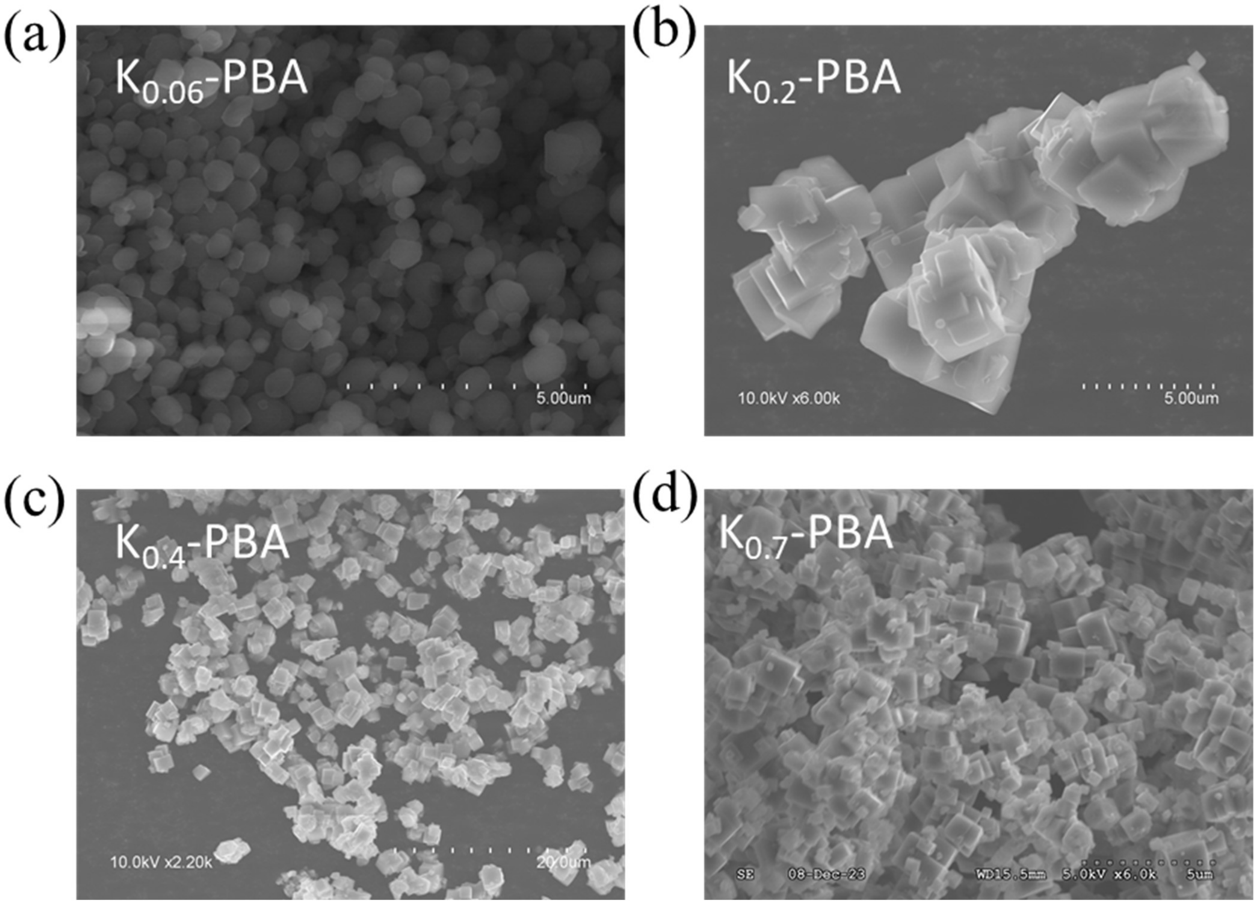

Figure S1. SEM images of the $K_{0.06}$-PBA (a), $K_{0.21}$-PBA (b), $K_{0.4}$-PBA (c), $K_{0.7}$-PBA (d) microcrystals and amount of K calculated from SEM elemental analysis.

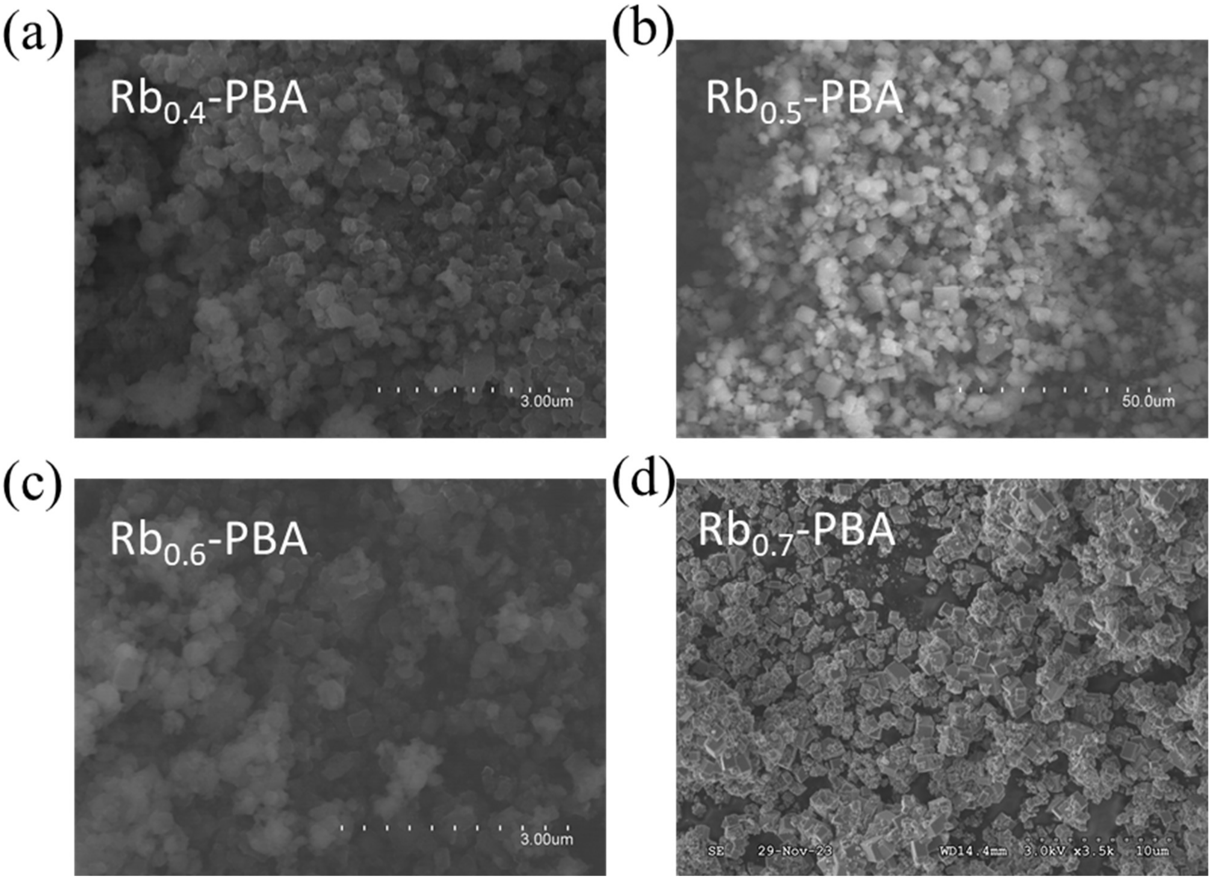

Figure S2. SEM images of the $Rb_{0.4}$-PBA (a), $Rb_{0.5}$-PBA (b), $Rb_{0.6}$PBA (c), $Rb_{0.7}$-PBA (d) microcrystals and amount of Rb calculated from SEM elemental analysis.

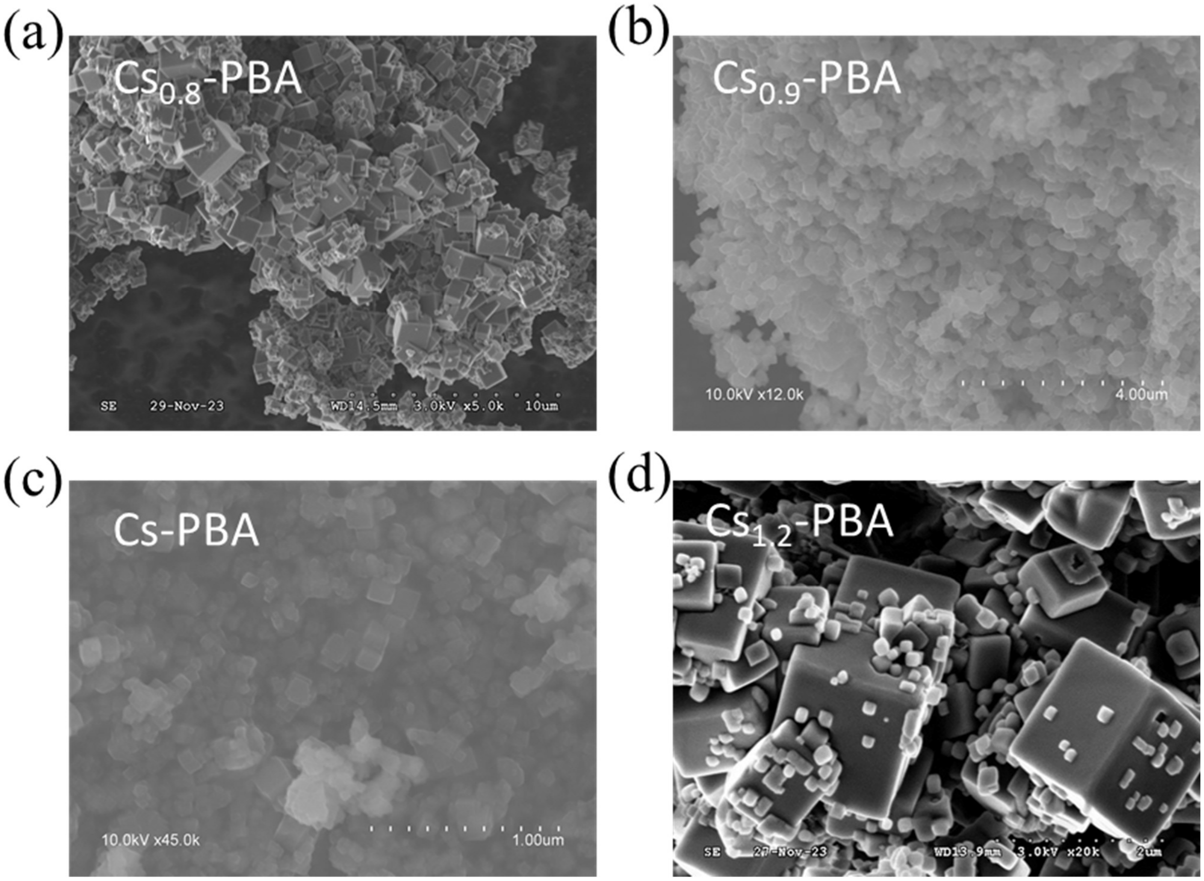

Figure S3. SEM images of the $Cs_{0.8}$-PBA (a), $Cs_{0.9}$-PBA (b), Cs-PBA (c), $Cs_{1.2}$-PBA (d) microcrystals and amount of Cs calculated from SEM elemental analysis.

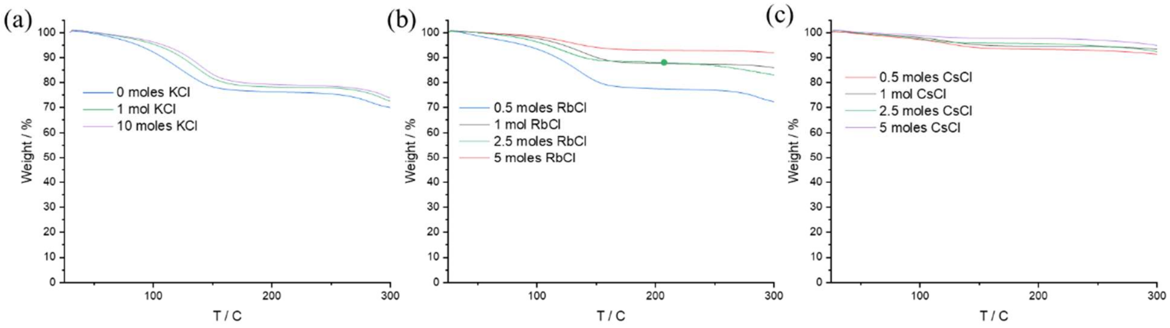

Figure S4. TGA analysis at air and 10 K/min of K_x-PBA (a), Rb_x-PBA (b), and Cs_x-PBA (c).

Table S1. Summary of the raw ICP-MS data extracted from the different samples.

	Initial ACl / moles	Initial $MnCl_2$ and $K_3[Fe(CN)6]$ / moles	A (mg/g)	Mn (mg/g)	Fe (mg/g)
K	0	0.5	17.5±0.9	231±5	166±3
	1		48.2±0.6	202±3	151±3
	10		115.8±1.7	162±3	125±2
Rb	0.5	0.5	119±6	217±9	168±7
	1		176±5	192±5	159±4
	2.5		203±2	166±2	149±2
	5		303±4	208±4	197±3
Cs	0.5	0.5	302±5	140±3	119±6
	1		308±11	131±3	119±6
	2.5		361±5	143±4	129±4
	5		343±2	132±2	112±8

Table S2. Summary of the synthetic concentration used for the A-PBA crystals and the molecular formula calculated from EDS, ICP-MS, TGA, and XRD Rietveld refinement.

	Initial ACl / moles	Initial $MnCl_2$ and $K_3[Fe(CN)6]$ / moles	EDS molecular formula	ICP molecular formula	XRD molecular formula
K	0	0.5	$K_{0.06}Mn[Fe(CN)_6]_{0.7}$	$K_{0.11}Mn[Fe(CN)_6]_{0.71}(H_2O)_{1.39}$	-
	0.5		$K_{0.2}Mn[Fe(CN)_6]_{0.8}$		-
	1		$K_{0.4}Mn[Fe(CN)_6]_{0.8}$	$K_{0.34}Mn[Fe(CN)_6]_{0.73}(H_2O)_{1.26}$	-
	5		$K_{0.5}Mn[Fe(CN)_6]_{0.8}$		-
	10		$K_{0.7}Mn[Fe(CN)_6]_{0.9}$	$KMn[Fe(CN)_6]_{0.76}(H_2O)_{1.15}$	$K_{0.91}Mn[Fe(CN)_6]_{0.80}$
Rb	0.5	0.5	$Rb_{0.4}Mn[Fe(CN)_6]_{0.8}$	$Rb_{0.35}Mn[Fe(CN)_6]_{0.76}(H_2O)_{1.24}$	-
	1		$Rb_{0.5}Mn[Fe(CN)_6]_{0.8}$	$Rb_{0.59}Mn[Fe(CN)_6]_{0.81}(H_2O)_{0.65}$	-
	2.5		$Rb_{0.6}Mn[Fe(CN)_6]_{0.8}$	$Rb_{0.79}Mn[Fe(CN)_6]_{0.88}(H_2O)_{0.70}$	-
	5		$Rb_{0.7}Mn[Fe(CN)_6]_{0.9}$	$Rb_{0.94}Mn[Fe(CN)_6]_{0.93}(H_2O)_{0.39}$	$Rb_{0.95}Mn[Fe(CN)_6]$
Cs	0.5	0.5	$Cs_{0.8}Mn[Fe(CN)_6]_{0.9}$	$Cs_{0.89}Mn[Fe(CN)_6]_{0.88}(H_2O)_{0.37}$	$Cs_{0.93}Mn[Fe(CN)_6]$
	1		$Cs_{0.9}Mn[Fe(CN)_6]_{0.9}$	$Cs_{0.97}Mn[Fe(CN)_6]_{0.89}(H_2O)_{0.35}$	-
	2.5		$CsMn[Fe(CN)_6]$	$Cs_{1.04}Mn[Fe(CN)_6]_{0.89}(H_2O)_{0.25}$	-
	5		$Cs_{1.2}Mn[Fe(CN)_6]$	$Cs_{1.07}Mn[Fe(CN)_6]_{0.84}(H_2O)_{0.13}$	-

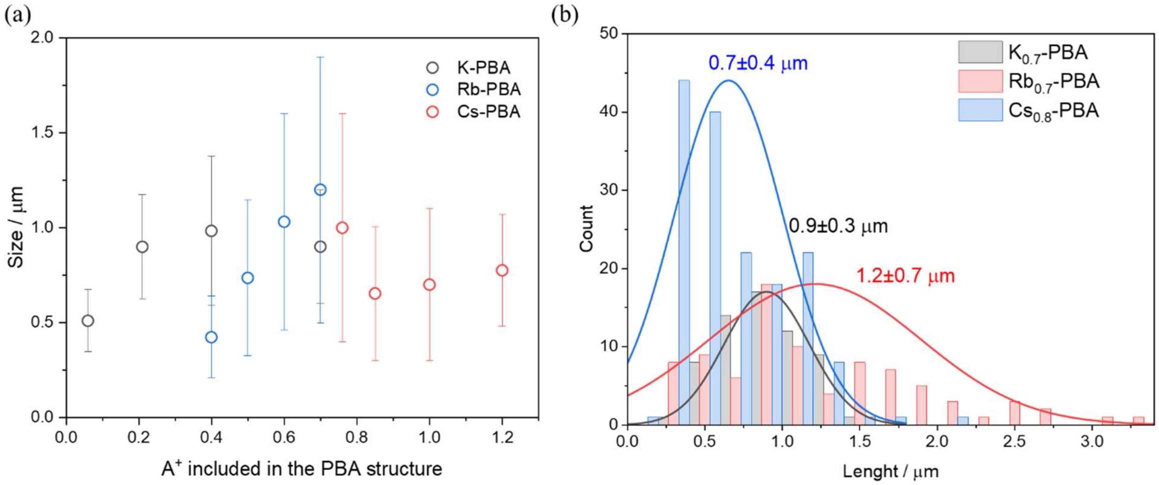

Figure S5. a) Size of the microcrystals calculated from SEM images as a function of the A^+ cation inclusion. (b) Size of the K-PBA, $Rb_{0.94}$-PBA, and $Cs_{0.89}$-PBA with their error.

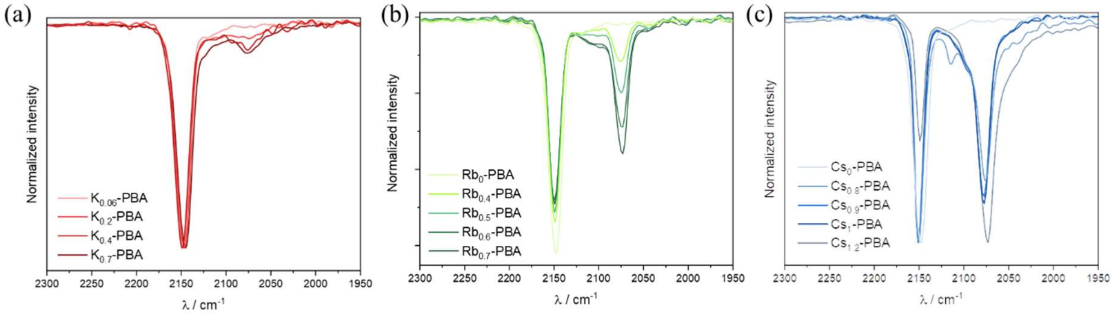

Figure S6.FT-IR spectra of the different A-PBA samples prepared with a different concentration of ACl. It must be noted that the molecular A content was stimated from EDS analysis.

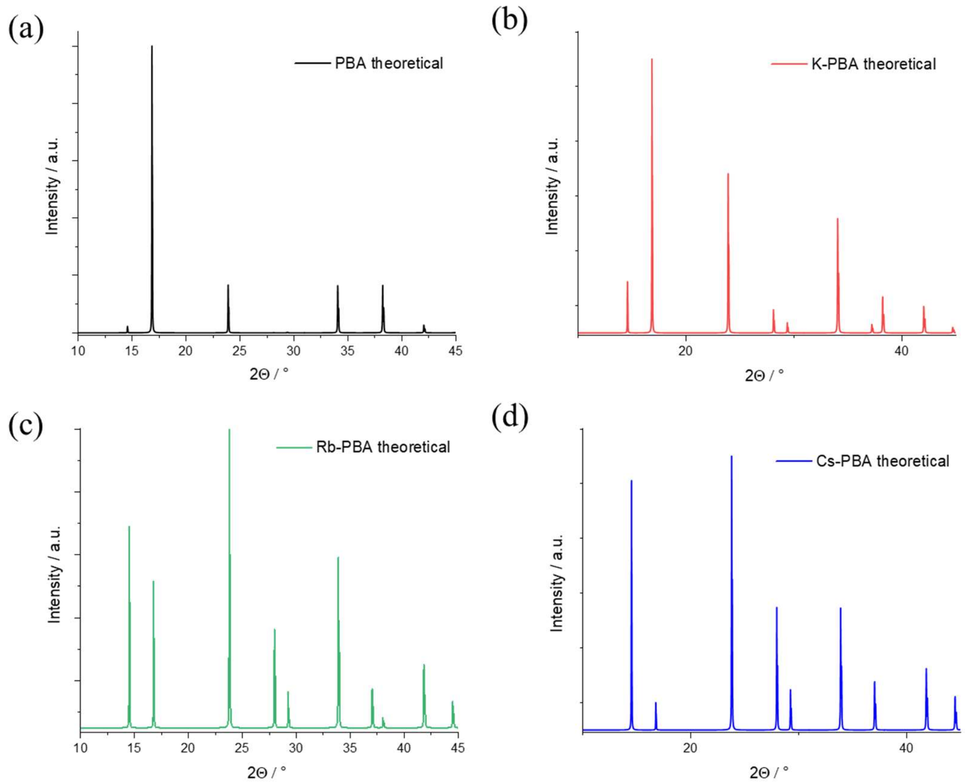

Figure S7. PXRD simulation spectra for the bare PBA (a), and perfects K-PBA (b), Rb-PBA (c), and Cs-PBA (d), using Cu K-α radiation.

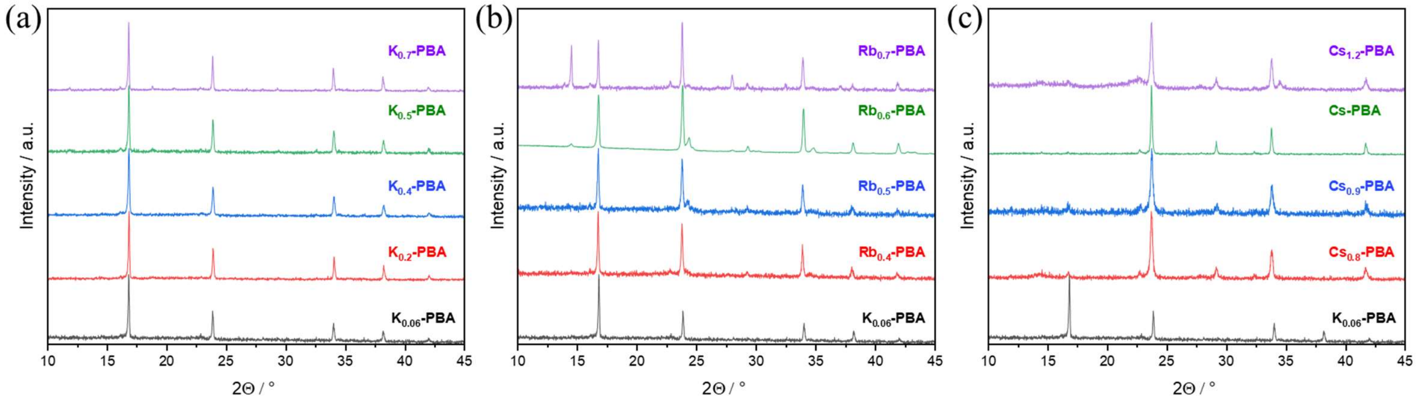

Figure S8. Experimental of the K_x-PBA (a), Rb_x-PBA (b), and Cs_x-PBA (c), depending on the amount of initial ACl determined by EDS analysis, measured with a Cu K-α source.

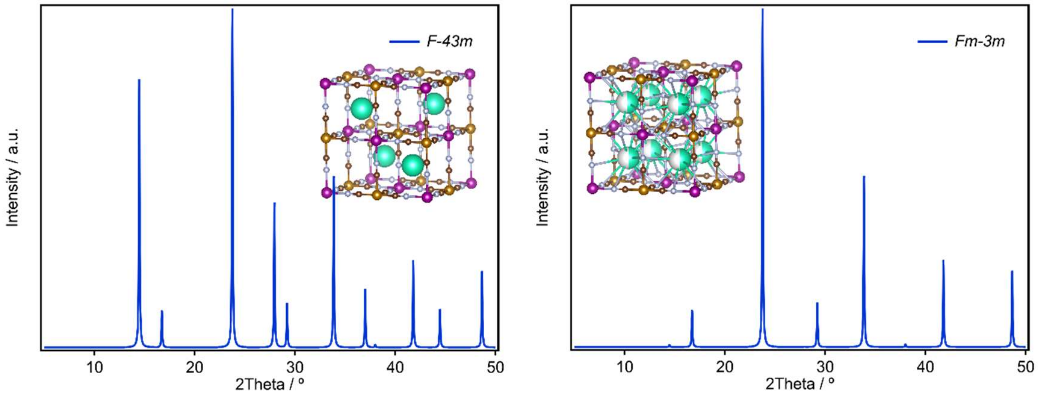

Figure S9 – Comparison of the simulated X-ray diffraction pattern for $CsMn[Fe(CN)_6]$ in space groups *F-43m* (left) and *Fm-3m* (right) showing how different arrangement of Cs ions inside the unit cell significatively impact the relative intensities

Rietveld Refinement

Analysis of the PXRD patterns via Rietveld refinement was carried out using the software TOPAS Academic v6.[23] Initially, $Rb_{0.94}$-PBA and $Cs_{0.89}$-PBA were refined in the *F-43m* space group. However, unlike $Rb_{0.94}$-PBA, no occupational A-site cation order was found in $Cs_{0.89}$-PBA, and thus it was better modelled in the *Fm-3m* space group. Similarly, K-PBA was found to crystallize in the primitive cubic unit cell, likely due to the ordering of the Fe vacancies in the material, but with no A-site cation ordering and it was thus modelled in the cubic space group *Pm-3m*. The atomic displacement parameters for the A-site (K, Rb or Cs), Mn, Fe and the CN ligands were refined independently, showing similar displacement parameters for Mn and Fe between samples but with important differences in the thermal displacement parameters of the CN ligands and A-site cations, which varied following the sequence: K-PBA > $Rb_{0.94}$-PBA > $Cs_{0.89}$-PBA. The final Rietveld refinement fits and data using the cubic structural models are displayed in Tables S3, S5, S7 and Figures S10-12. ISODISTORT[21] was used to lower the symmetry of the parent cubic unit cells to a rhombohedral unit cell. This allowed us to refine the displacement of the CN ligands from their ideal positions in the cubic structure as a function of the A-site cation. To avoid overfitting, only the tilt modes corresponding to the primary order parameters Γ_4^+ and Γ_5^+ were refined. This resulted in variable tilting of the MnN_6 and FeC_6 octahedra as well as in a lowering of the thermal displacement of the CN ligand from 3.69(9) Å^2 to 1.32(5) Å^2, in K-PBA, and from 2.76(11) Å^2 to 1.51(5) Å^2, in $Rb_{0.94}$-PBA. Little difference was observed for the atomic displacement parameters obtained of the CN ligand between the cubic and distorted models for $Cs_{0.89}$-PBA. At this point, all Mn, Fe, C and N atoms were found to have similar displacement parameters, so they were constrained to the same value in order to reduce the number of free parameters. This produced equivalent Rietveld fits as those obtained using the cubic models, but it also allowed us to estimate the deviation of the CN ligands from linearity as a function of the A-site cation.

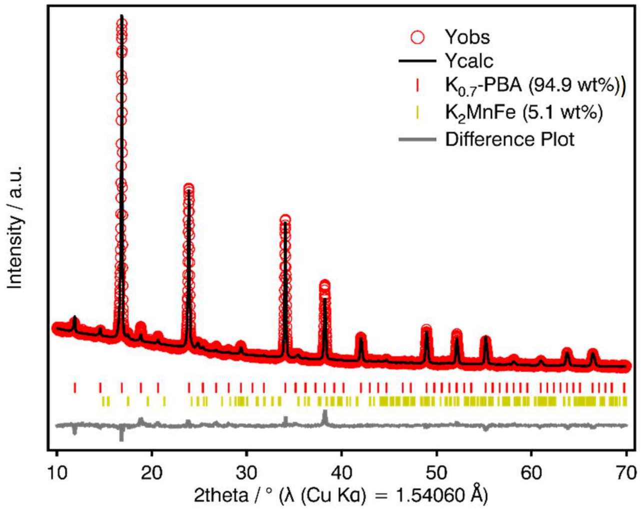

Figure S10 – Rietveld refinement fit for the K-PBA at ambient conditions in the cubic space group *Pm-3m*. Yellow tick marks correspond to the $K_2Mn[Fe(CN)_6]$ impurity (5.1 wt %)

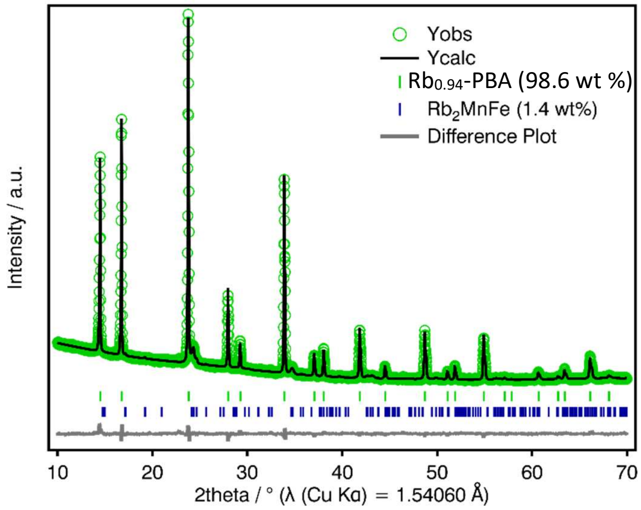

Figure S11 – Rietveld refinement fit for the $Rb_{0.94}$-PBA at ambient conditions in the cubic space group *F-43m*. Blue tick marks correspond to the $Rb_2Mn[Fe(CN)_6]$ impurity (1.4 wt %)

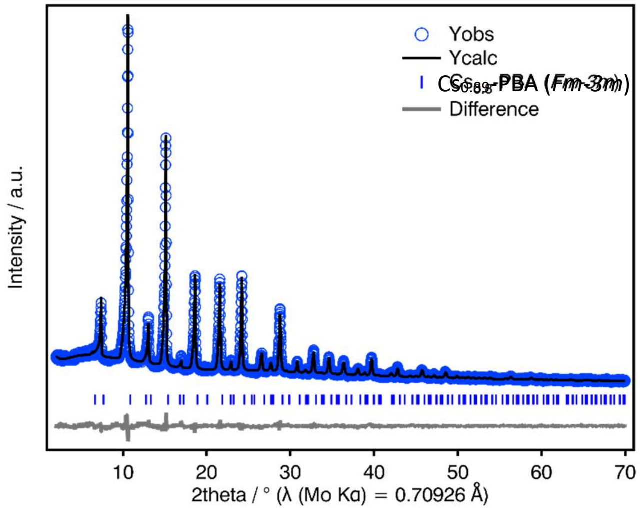

Figure S12 – Rietveld refinement fit for the $Cs_{0.89}$-PBA at ambient conditions in the cubic space group *Fm-3m*.

Table S3 – Crystallographic details from the Rietveld refinement of the cubic K-PBA structure.

	K-PBA
Space group	Pm-3m
a, Å	10.5197(2)
V, Å^3	1164.16(5)
Z	4
Temperature, K	298
Wavelength, Å	1.54060/1.54449 (Cu Kα1/Kα2)
2Θ range, °	10-80
Rp, %	1.84
Rwp, %	2.51

**Element**	**Wyckoff position**	**Atomic fractional coordinates**			**Occ**	**B_{iso} / Å^2**
		**x / a**	**y / b**	**z / c**		
Mn	1a	0	0	0	1.0	0.85(5)
Mn	3c	0	1/2	1/2	1.0	
Fe	1b	1/2	1/2	1/2	0.356(6)	0.85(5)
Fe	3d	1/2	0	0	0.951(4)	
K	8g	1/4	1/4	1/4	0.456(2)	12.8(2)
C	6e	0.3108	0	0	0.951(4)	3.69(9)
N	6e	0.2005	0	0	0.951(4)	3.69(9)
O	6e	0.2100	0	0	0.049(4)	3.69(9)
C	6f	0.3108	1/2	1/2	0.356(6)	3.69(9)
N	6f	0.2005	1/2	1/2	0.356(6)	3.69(9)
O	6f	0.2100	1/2	1/2	0.644(6)	3.69(9)
C	12h	0.1887	1/2	0	0.951(4)	3.69(9)
N	12h	0.2995	1/2	0	0.951(4)	3.69(9)
O	12h	0.2900	1/2	0	0.049(4)	3.69(9)

Table S4 – Crystallographic details from the Rietveld refinement of the distorted K-PBA structure

Distorted K-PBA	
Space group	R-3
a, Å	14.8754(6)
a, Å	18.226(2)
V, Å³	3492.8(5)
Z	12
Temperature, K	298
Wavelength, Å	1.54060/1.54449 (Cu Kα1/Kα2)
2Θ range, °	10-80
Rp, %	1.79
Rwp, %	2.44

**Element**	**Wyckoff position**	**Atomic fractional coordinates**			**Occ**	**B_{iso} / Å^2**
		**x / a**	**y / b**	**z / c**		
Mn	3a	0	0	0	1.0	1.32(5)
Mn	9e	1/2	0	0	1.0	
Fe	3b	0	0	1/2	0.356(6)	1.32(5)
Fe	9d	1/2	0	1/2	0.951(4)	
K	6c	0	0	3/4	0.456(2)	12.6
K	18f	1/6	1/3	1/12	0.456(2)	12.6
C	18f	0.783(3)	0.5405	0.2297	0.951(4)	1.32(5)
N	18f	0.748(3)	0.4670	0.2665	0.951(4)	1.32(5)
O	18f	0.751(3)	0.4733	0.2633	0.049(4)	1.32(5)
C	18f	0.2702	0.5405	0.2297	0.356(6)	1.32(5)
N	18f	0.261(2)	0.4670	0.2665	0.356(6)	1.32(5)
O	18f	0.264(2)	0.4733	0.2633	0.644(6)	1.32(5)
C	18f	0.907(4)	0.295(2)	0.107(3)	0.951(4)	1.32(5)
N	18f	0.931(3)	0.371(2)	0.071(2)	0.951(4)	1.32(5)
O	18f	0.928(4)	0.364(5)	0.075(2)	0.049(4)	1.32(5)
C	18f	0.782(4)	0.738(3)	0.232(2)	0.951(4)	1.32(5)
N	18f	0.752(3)	0.771(2)	0.281(2)	0.951(4)	1.32(5)
O	18f	0.756(3)	0.767(2)	0.278(2)	0.049(4)	1.32(5)

Table S5– Crystallographic details from the Rietveld refinement of the cubic $Rb_{0.94}$-PBA structure.

$Rb_{0.94}$-PBA	
Space group	F-43m
a, Å	10.56393(8)
V, Å^3	1178.90(3)
Z	4
Temperature, K	298
Wavelength, Å	1.54060/1.54449 (Cu Kα1/Kα2)
2Θ range, °	10-70
Rp, %	1.21
Rwp, %	1.58

**Element**	**Wyckoff position**	**Atomic fractional coordinates**			**Occ**	**B_{iso} / Å^2**
		**x / a**	**y / b**	**z / c**		
Mn	4a	0	0	0	1.0	1.51(5)
Fe	4b	1/2	1/2	1/2	1.000(9)	1.51(5)
Rb	4c	1/4	1/4	1/4	0.935(4)	7.3(1)
Rb	4d	3/4	3/4	3/4	0.017(1)	7.3(1)
C	24f	0.3179(15)	0	0	1.000(9)	2.8(1)
N	24f	0.2053(12)	0	0	1.000(9)	2.8(1)

Table S6 – Crystallographic details from the Rietveld refinement of the distorted $Rb_{0.94}$-PBA structure.

Distorted $Rb_{0.94}$-PBA	
Space group	R3
a, Å	7.4694(2)
c, Å	18.300(1)
V, Å³	884.15(8)
Z	3
Temperature, K	298
Wavelength, Å	1.54060/1.54449 (Cu Kα1/Kα2)
2Θ range, °	10-70
Rp, %	1.23
Rwp, %	1.60

**Element**	**Wyckoff position**	**Atomic fractional coordinates**			**Occ**	B_{iso} **/ Å²**
		**x / a**	**y / b**	**z / c**		
Mn	3a	0	0	0	1.0	1.51(5)
Fe	3a	0	0	1/2	1.0	1.51(5)
Rb	3a	0	0	3/4	0.935(4)	7.3
Rb	3a	0	0	1/4	0.017(1)	7.3
C	9b	0.4389(9)	0.9093	0.2273	1.0	1.51(5)
N	9b	0.5413(8)	0.0596	0.2649	1.0	1.51(5)
C	9b	0.4389(9)	0.4240	0.1060	1.0	1.51(5)
N	9b	0.1313(9)	0.2855	0.0625	1.0	1.51(5)

Table S7 – Crystallographic details from the Rietveld refinement of the cubic $Cs_{0.89}$-PBA structure.

$Cs_{0.89}$-PBA	
Space group	Fm-3m
a, Å	10.5768(3)
V, Å³	1183.2(1)
Z	4
Temperature, K	298
Wavelength, Å	0.70926 (Mo Kα1)
2Θ range, °	2-74
Rp, %	4.63
Rwp, %	5.97

**Element**	**Wyckoff position**	**Atomic fractional coordinates**			**Occ**	**B_{iso} / Å²**
		**x / a**	**y / b**	**z / c**		
Mn	4a	0	0	0	1.0	0.72(4)
Fe	4b	1/2	1/2	1/2	1.00(1)	0.72(4)
Cs	4c	1/4	1/4	1/4	0.467(2)	4.37(7)
C	24f	0.318(9)	0	0	1.00(1)	1.09(9)
N	24f	0.210(7)	0	0	1.00(1)	1.09(9)

Table S8 – Crystallographic details from the Rietveld refinement of the distorted $Cs_{0.8}$-PBA structure.

	Distorted $Cs_{0.89}$-PBA
Space group	R-3
a, Å	7.4796(9)
c, Å	18.316(4)
V, Å³	887.4(4)
Z	3
Temperature, K	298
Wavelength, Å	0.70926 (Mo Kα1)
2Θ range, °	2-74
Rp, %	4.54
Rwp, %	5.86

**Element**	**Wyckoff position**	**Atomic fractional coordinates**			**Occc**	**B_{iso} / Å²**
		**x / a**	**y / b**	**z / c**		
Mn	3a	0	0	0	1.0	0.72(4)
Fe	3b	0	0	1/2	1.0	0.72(4)
Cs	6c	0	0	3/4	0.467(2)	4.36(6)
C	18f	0.467(3)	0.9087	0.2272	1.0	0.72(4)
N	18f	0.551(2)	0.0536	0.2634	1.0	0.72(4)

Extended X-ray Absorption Fine Structure (EXAFS)

A qualitative comparison of Fourier Transforms of Fe K-edge EXAFS spectra shows that the local structure around Fe atoms in all samples is similar with small differences around the third coordination shell. The quantitative information on Fe local neighbourhood in the samples was obtained from quantitative EXAFS analysis. EXAFS spectra were modelled with ab-initio FEFF[1] calculation using the simultaneous fit of the three spectra. Models include three coordination shells, comprising all single-scattering and significant multiple-scattering paths of the photoelectron up to 5.7 Å. Structure parameters of the nearest neighbours of Fe atoms are listed in the Table S9.

In the FEFF models for each coordination shell, the distance from the absorbing atom and Debye-Waller factor were allowed to vary in the fit, while the number of neighbours was fixed to the values defined in crystallographic data. The amplitude reduction factor and common shift of energy origin for all scattering paths were allowed to vary in the fit for each spectrum. In order to stabilize the fit by increasing the number of independent points, Debye-Waller factors for each coordination shell in the simulation relaxation were constrained to the common values for the three samples. In addition, the common shift of energy origin and amplitude reduction factor was also constrained to the common values in the fit for the three samples. A very good agreement between EXAFS model and experimental spectra of the three samples was found in the R-range of [1.0 Å – 5.1 Å] and in the k-interval of [2.1 $Å^{-1}$ - 14.5 $Å^{-1}$] using k^3-weight (Figure S13).

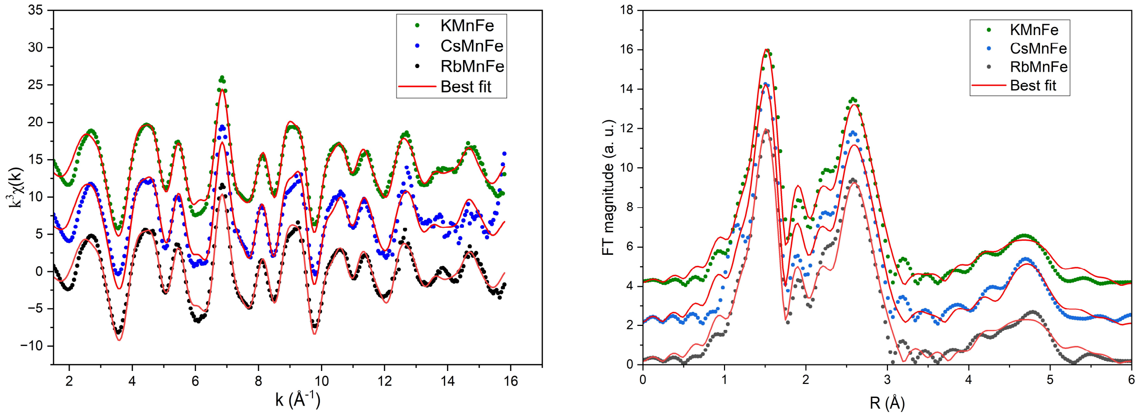

Figure S13. Fe K-edge EXAFS spectra (left) and corresponding Fourier Transform magnitudes (right) of the K-PBA (a), $Rb_{0.94}$-PBA, and $Cs_{0.89}$-PBA. The film is plotted as χ (R) and calculated with k weight of 3. Circles represent experimental data, and the red solid line the best fit EXAFS model.

Table S9. EXAFS Fitting atomic distances of the different samples.

	Bond	R / Å^2	σ^2 / Å^2
K-PBA	Fe-Mn	**5.314 ± 0.009**	0.0169 ± 0.0011
	Fe-C	1.930 ± 0.002	0.0033 ± 0.0003
	Fe-N	3.135 ± 0.019	0.0057 ± 0.0004
	Fe - K	4.688 ± 0.058	0.0201 ± 0.0082
	Fe – N(2)	5.719 ± 0.020	0.0065 ± 0.0046
$Rb_{0.94}$-PBA	Fe- Mn	**5.326 ± 0.020**	0.0169 ± 0.0011
	Fe-C	1.924 ± 0.005	0.0033 ± 0.0003
	Fe-N	3.131 ± 0.027	0.0057 ± 0.0004
	Fe-Rb	4.513 ± 0.112	0.0176 ± 0.0134

	Fe – N(2)	5.711 ± 0.049	0.0065 ± 0.0046
$Cs_{0.89}$-PBA	Fe- Mn	**5.316 ± 0.035**	0.0169 ± 0.0011
	Fe-C	1.921 ± 0.008	0.0033 ± 0.0003
	Fe-N	3.132 ± 0.036	0.0057 ± 0.0004
	Fe-Cs	4.879 ± 0.087	0.0125 ± 0.0088
	Fe – N(2)	5.738 ± 0.076	0.0065 ± 0.0046

Pair distribution functions

Total scattering data was collected in transmission mode in a STOE Stadi-P diffractometer equipped with a Mo source (λ = 0.70926 Å, curved Ge monochromator) and a Mythen 2 DCS4 detector in the 2theta range 2-140° using 0.7 mm borosilicate glass capillaries and an overall data collection time of 15h. The data was corrected for background, Compton scattering, and detector effects and Fourier transformed to G(r) (Q_{max} = 16.5 $Å^{-1}$) with a Lorch function using the software PDFGetX2[2]. Instrumental parameters were determined by measuring a LaB_6 standard under identical conditions (Figure S15).

To investigate the local structure of the K-PBA and $Cs_{0.89}$-PBA samples, we collected PDF data using and a STOE Stadi-P diffractometer equipped with a Mo source. Figure S14 displays the comparison between the PDFs of K-PBA and $Cs0_{.89}$-PBA samples showing differences in the Fe-Mn distance at ca. 5.3-5.4 Å being larger for the Cs sample. In order to estimate the extent of the distortion due to the size reduction of the A-site cation, we estimated the average M-C-N and M-N-C angle by taking into account the average M-C/M-N (2.12 Å) and Mn-Fe distances (5.315 and 5.379 Å for K-PBA and $Cs_{0.89}$-PBA, respectively) as obtained from the PDF by fitting the peaks with Gaussian functions. The estimation of the degree of distortion (α) was done assuming a transverse distortion of the C-N ligands, as observed previously in the $Fe[Fe(CN)_6]$, and a C-N bond distance of 1.15 Å, although we note that other type of distortions may be present. This yielded M-CN angles of 157º and 171º for K-PBA and $Cs_{0.89}$-PBA, respectively, in good agreement with our XRD analysis, further suggesting that the smaller ions K^+ ions promote the distortion of the CN ligands due to stronger electrostatic interactions.

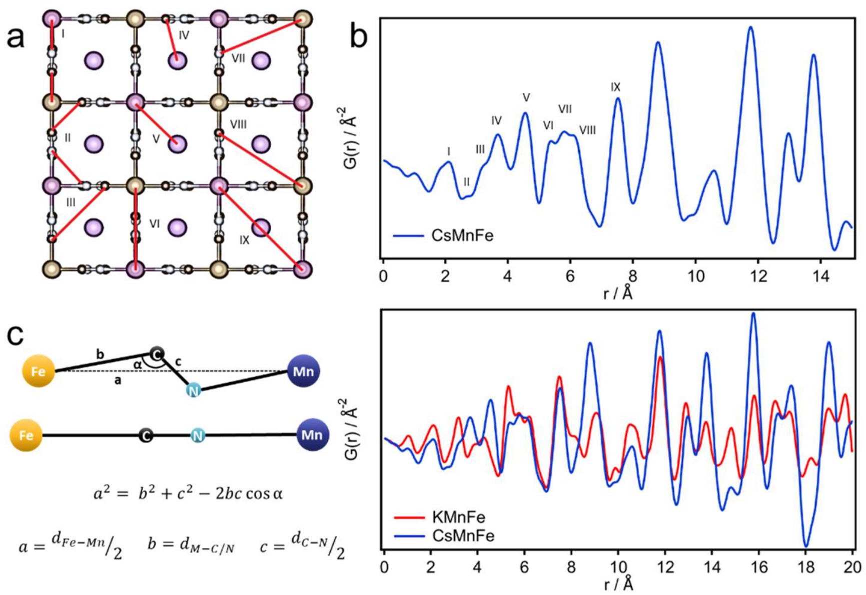

Figure S14. a) scheme of the structure and distances observed in the Pair distribution functions of pigments Cs0.89-PBA.b) K-PBA, red, and Cs0.89-PBA, blue Refinement of the pair distribution function, G(r). c) Geometrical model used to calculate the tilting.

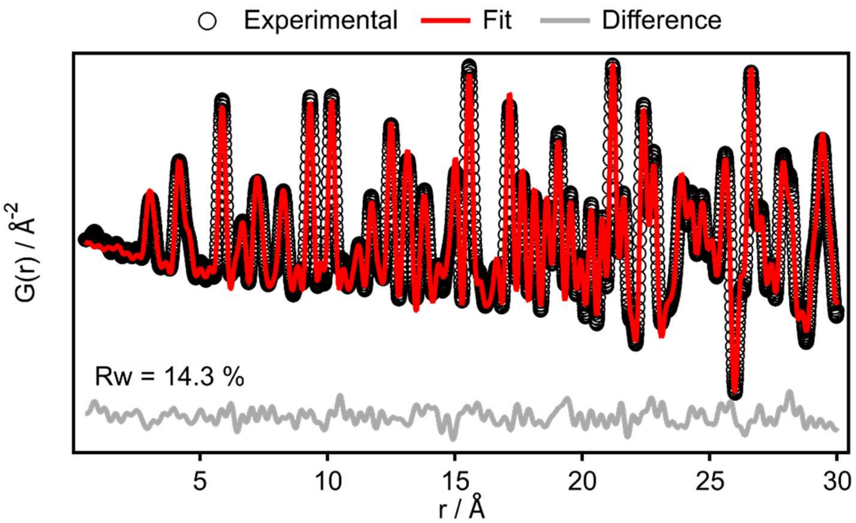

Figure S15. PDF fit of the experimental G(r) for the LaB_6 standard carried out using PDFGui software.[3]

Magnetic data

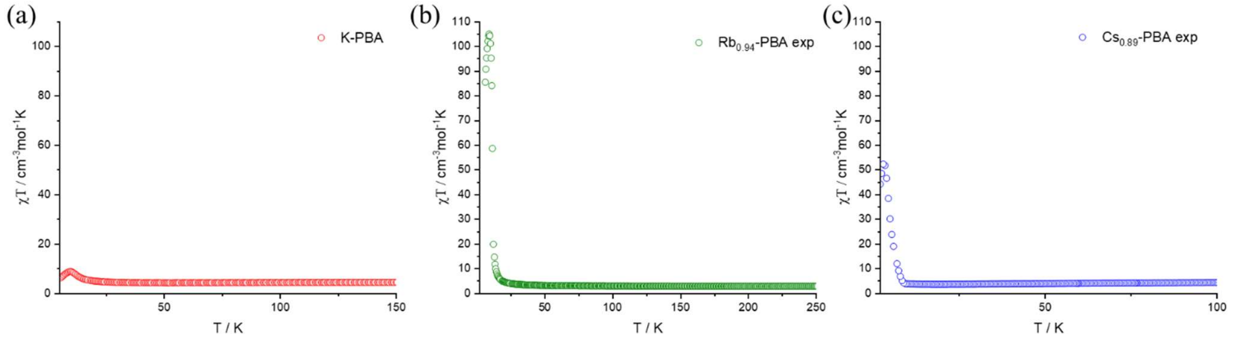

Figure S16. χT as a function of the temperature for K_x-PBA (a), Rb_x-PBA (b), and Cs_x-PBA (c).

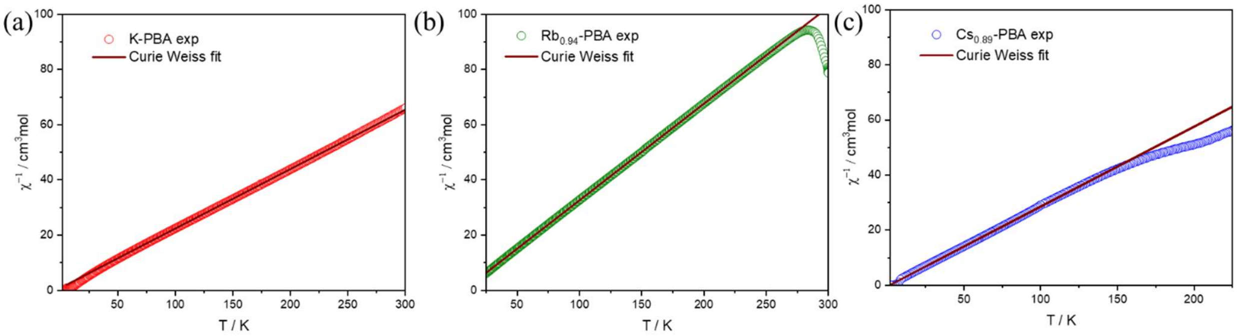

Figure S17. 1/χ as a function of the temperature for K_x-PBA (a), Rb_x-PBA (b), and Cs_x-PBA (c). The experimental data are represented with empty spheres and the fitting with straight lines.

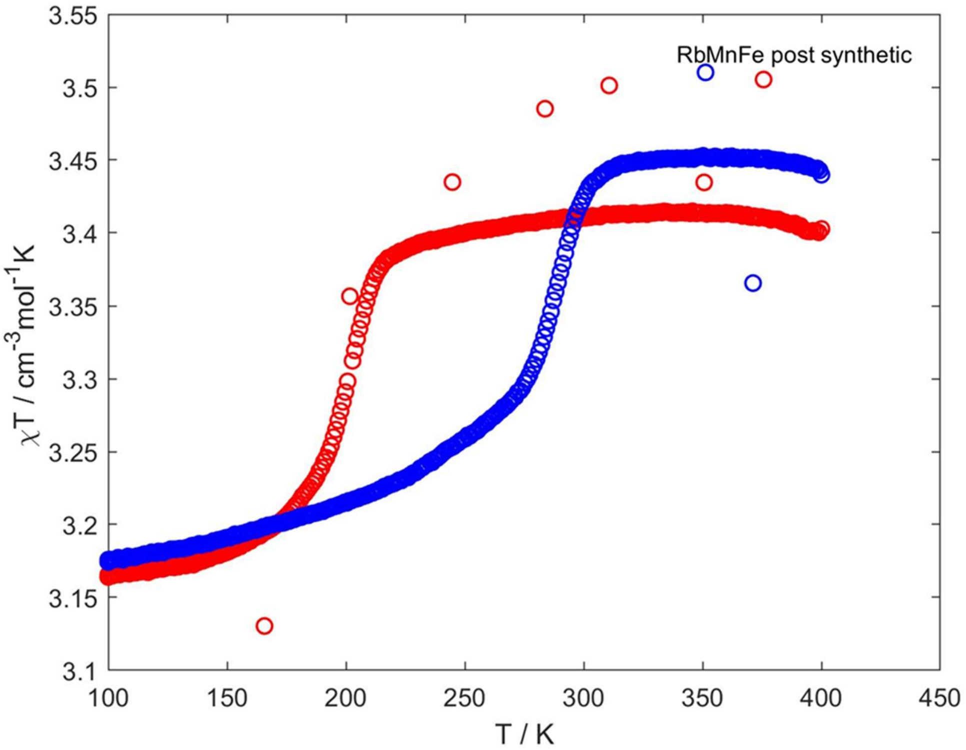

Figure S18. χT as a function of the temperature for the $K_{0.7}$-PBA after the K exchange for Rb. In blue the heating, and in red the cooling.

References

[1] J. J. Rehr and R. C. Albers, Rev. Mod. Phys. 72, 621, (2000)

[2] Qiu, X.; Thompson, J. W.; Billinge, S. J. L. J. Appl. Crystallogr. 2004, 37, 678

[3] C. L. Farrow, P. Juh´as, J. W. Liu, D. Bryndin, E. S. Boˇzin, J. Bloch, Th. Prof-

fen and S. J. L. Billinge, PDFfit2 and PDFgui: computer programs for studying nanostructure in crystals. J. Phys.: Condens. Matter, 19, 335219 (2007)
